# India’s Conception of Community Data and Addressing Concerns for Access to Justice

**DOI:** 10.1007/s44206-024-00102-5

**Published:** 2024-03-23

**Authors:** Siddharth Peter de Souza, Kritika Bhardwaj

**Affiliations:** 1 Tilburg Law School, Tilburg University, Tilburg, The Netherlands; 2 African Centre for Epistemology and Philosophy of Science, University of Johannesburg, Johannesburg, South Africa; 3 Supreme Court of India, New Delhi, India

**Keywords:** Non personal data, Community data, Data justice, Access to justice

## Abstract

This paper discusses the idea of community data that was introduced in the Non-Personal Data framework in India. Our interest is to engage with existing commentaries on the definitional challenges around who is a community, how it is constituted, who it represents, as well as propose a framework to be able to explore how to address concerns of access to justice. In our attempt to offer a model to operationalise community data, we argue that such community data includes three crucial aspects, that is, the identification of belonging with a community, the capacity to participate within a community, and finally opportunity to exit the community. Consequently, justice in terms of access to, and use of community data inherently includes an analysis of the individual’s standing in the community.

## Introduction

There is growing acknowledgment of the fact that the focus of big data analytics has shifted from profiling individuals to predicting behaviours of communities (Taylor et al., 2017b). These community insights in turn form the basis for commercial or policy decisions impacting that group. The biometric identification project in India (Aadhaar) is an example which has demonstrated the variegated effects that the project has had in terms of communities. These include being excluded for reasons such as challenges of technology hardware, the impacts of local middlemen in ensuring distribution of services in specific regions or because of the inherent unreliability of biometrics as a tool for identification itself (Khera, 2015). For instance, those engaged in manual labour were often denied their entitlements because their fingerprints could not be easily authenticated owing to the nature of their work (Ramanathan, 2016). These examples showcase that Aadhaar as a socio-technical system has resulted in people being marginalised by technology in different ways, deeply influenced by their socio-political and economic conditions (Ramanathan, 2011).

Aadhaar is an example of how data driven systems can exacerbate existing structural injustices (boyd & Crawford, 2012) on account of their collection methods, the ways in which databases and data sets are structured or the categories that are used to highlight certain types of issues over others (Balayn & Gürses, 2021). It demonstrates how the experiences of members of a community will differ on account of issues related to identity and interests. Accordingly, the lived experience of members of a community on account of these factors need to be accounted for in interactions with data driven systems (Bokil et al., 2021).

Recognition of a collective interest is imperative in seeking redressal in such instances where the impact is dispersed across a community (Arora, 2016), and layered, unequal, and varied in terms of how they affect its members (Milan & Treré, 2019). The recognition of collective interest, and the potential for collective harms, has in many ways inspired the push towards conceptualising what community rights to data should be, and how to operationalise them.

India has seen a growing policy push towards harnessing data for public and entrepreneurial purposes in recent times (Aayog, 2018). There are several reasons for this. First, there have been rapid advancements in data gathering and storage capabilities at reduced costs (Economic Survey of India, 2019). Second, developments in the field of artificial intelligence and machine learning have revolutionised how gathered data can be used, and the insights it offers. Third, large scale aggregation and marshalling of personal data by a handful of technological platforms has demonstrated how accumulation of personal data can be exploitative and detrimental to both individual rights and market competition (“Big Tech’s Heavy Hand Around the Globe”, 2020; Lopez Solano et al., 2022; Sharon, 2020).

However, despite the recent push towards recognition of community rights over data, existing policy articulations in India stop short of identifying the basis for membership to a community, and the nature and scope of data which the community would be entitled to exercise its rights over.

In this paper, we examine the proposals to define community data in India and argue for the value of recognising community data. In doing so, we examine how the law sees communities and communities see the law to ascertain ways to operationalise a model for community data. This is important because the extant legal regime in India already contains useful instruments to not only account for differentiated identities and interests, but also for avenues that allow for such interests to be expressed and addressed. We will discuss two particular cases, that of minority rights, and those of public interest litigation.

This paper analyses developments in data governance in India as a case study and offers pathways to the governance of data communities. In its attempt to offer a model to operationalise community data, this paper proposes to unpack how to ensure access to (community data) justice. We argue that such community data justice includes three crucial aspects, that is, the identification of belonging with a community, the capacity to participate within a community, and finally opportunity to exit the community. Consequently, justice in terms of access to, and use of community data inherently includes an analysis of the individual’s standing vis-à-vis the community.

The paper is structured as follows. The next section examines the proposal for community data in India tracing how it has developed, and why it is important to care for a community perspective. The third section discusses concerns and limitations with the proposed framework for community data to examine whether it addresses injustices in the governance of data. In the fourth section, the paper discusses how the law categorises and regulates communities in India through examples from other domains of law, with a view to examine whether these existing articulations offer any useful takeaways or lessons for operationalising community rights over data. In the fifth section, the paper proposes a model for access to (community data) justice.

## Why Should We Care About Communities—The Proposal for Community Data in India

The Indian state’s response to developments in the data economy has been to increasingly articulate data as a public good. In the Economic Survey of 2018–2019, the Indian government pitched for data to be recognised and utilised as a public good with the intention that data is “of the people, by the people and for the people” (Economic Survey of India, 2019). The Survey noted that anonymised data has tremendous social value which was not being adequately harnessed by the private sector because it was driven by narrower economic incentives as compared to a broader goal for social welfare (Economic Survey of India, 2019). The Survey advocates for citizens becoming equal beneficiaries of harnessed data in addition to governments and the private sector.

Around the same time, in a 2018 Report, a Committee of experts constituted to deliberate on a data protection legislation for India noted that non-personal data and emerging processing activities “hold considerable strategic or economic interest for the nation” and therefore merited further consideration (A free and fair digital economy protecting privacy, empowering indians committee of experts under the chairmanship of justice B.N. Srikrishna, n.d., p. 19). A manifestation of this approach was also seen in the draft bill on data protection released in 2019. While ostensibly for the regulation of personal data, the 2019 bill envisaged a framework which would have allowed the government to direct entities gathering data to disclose anonymised or other non-personal data for better targeting of services or formulation of evidence-based policies, in accordance with future government policy (“The personal data protection bill,” 2019, n.d.). The 2019 bill was subsequently referred to a Joint Parliamentary Committee (JPC). In its final report published in December 2021, the JPC fully endorsed the government’s power to pass such directives, noting that “data is an asset of national importance which is waiting to be tapped comprehensively” (SFLC, n.d.). In 2022, the Indian government ultimately withdrew the 2019 bill, citing the need for a “comprehensive legal framework” for the “digital ecosystem” generally (“Data protection bill: Government withdraws data protection bill”, n.d.).

In 2023, the Indian Parliament passed the Digital Personal Data Protection Act, 2023, a legislation “to provide for the processing of digital personal data in a manner that recognises both the right of individuals to protect their personal data and the need to process such personal data for lawful purposes and for matters connected therewith or incidental thereto”. Contrary to earlier drafts, the Act does not mention or seek to regulate non-personal data. Instead, the Indian government has indicated that it will introduce regulations for non-personal data under a reformed omnibus legislation for all digital services (Digital India law to frame rules for non-personal data sharing, n.d.).

Against this background, a discussion on the approaches to, and forms of regulation of non-personal data has become even more urgent (Gurumurthy & Chami, 2022; Sinha & Basu, 2021). A particularly important feature in some of the proposals for governance of non-personal data is the creation of a framework for community rights over data. These rights are envisaged as a community’s right to derive economic value and a right to minimise harms to itself which arise out of processing of data (Report by the committee of experts on non-personal data governance framework, 2020).

The most substantial articulation of community rights over data can be found in a 2020 report published by a Committee of experts constituted by the Indian government to deliberate on a framework for the regulation of non-personal data in 2019. The Committee released an initial report in July 2020, and after receiving feedback, a subsequent report in December 2020 (NPD Report).

Unlike the Economic Survey, the NPD Report does not automatically categorise all non-personal data as a public good. The NPD Report identifies the following principles as informing its approach to the regulation of non-personal data (NPD Report, p. 5):

1. Sovereignty: “India has rights over data of India”, over data created by Indian users and organisations.
2. Benefits of data must be passed on to the state as well as its people.
3. To facilitate innovation and contribute to growth within India and globally.
4. Ensure privacy.
5. Ensure regulations are simple and accessible.
6. Data should be freely available for innovation and entrepreneurship in India.

Significantly, the Committee does not identify any legal or other basis for the state to have sovereign rights over non-personal data. Instead, it focuses on the supposed benefits of such a regulatory regime, arguing that it will enable the state to unlock the economic benefits from non-personal data for itself as well as for citizens and communities.

With respect to its framework for community rights over data in particular, the underlying rationale for recognising such rights appears to be a need to vest the community with benefits of the data that it helped produce. In the Report, the Committee defines a community as “any group of people that are bound by common interests and purposes and involved in social and/or economic interactions” (NPD Report, p. 16). While providing illustrations, the report also envisages (but does not define), an “entirely virtual community”.

The NPD Report identifies two overarching rights that a community may be able to exercise over non-personal data. These are the right to derive economic value from the data, and a right to eliminate or minimise harms to the community (NPD Report, p. 16). These rights are proposed to be operationalised through identification of certain “High Value Datasets”, that is, datasets which are of economic or social value to the society generally. The Report envisages High Value Datasets as datasets which may be useful in policymaking, improving access to public services, useful for research etc. The creation, maintenance and sharing of High Value Datasets is proposed to be entrusted to a “data trustee”—a government or non-profit private organisation which will owe a “duty of care” to the community in relation to non-personal data associated with it (NPD Report, p. 18). Presumably with a view to ensure that the data trustee represents the community’s actual interests, the Report suggests that a data trustee must have the support of a minimum number of community constituents.

## Challenges and Limitations of the Definition of Community Data in the Indian Context from a Justice Standpoint

The proposals put forward to govern community data in India through the NPD Report raise several interesting challenges. This section explores some of the criticisms in particular from how far these proposals secure justice for communities. Justice in this instance, is understood both in terms of the institutions that enable justice, and also in terms of how justice is experienced as a result of socio-political realities.

The definition adopted by the Committee is extremely broad. The Committee proposes rights over data for any group of people with a common interest or purpose and who are joined by their social or economic interactions. The Committee’s express reference to “entirely virtual communities” is also encouraging. Recycling of data for different purposes and aggregating it with newer data may offer newer insights, giving rise to constantly evolving algorithmic groups—or virtual communities—which might have a common interest in access to and use of such data.

However, such a broad definition leads firstly to the problem of consent. Sinha and Basu argue that individuals who come to form an algorithmic community do not necessarily consent to being treated as such and therefore, to treat such a group as a community would be contrary to its members’ decisional autonomy, which is their ability to act according to their own interests or ideologies in the face of pressures from the community (Sinha & Basu, 2021). They contrast such groups or communities from “tight knit communities” such as “farming communities” or “indigenous groups”.

Secondly, there is a problem of delineating boundaries when it comes to communities. For instance, identifying a “tight knit community” may not always be straightforward either. Taking the illustration of a “farming community”, it is not clear what the geographical limits of such a community are, or if it includes members who might have common interests based on climate concerns, market access, labour regulations, or the produce they grow, irrespective of their geography. Depending on the data in question, there is merit in identifying different kinds of farming communities. Purtova has argued to examine data as a systems resource, which includes people, platforms, and data. Taking such an approach acknowledges that there is a data ecosystem that exists which is based on the interactions and relations between people and data (Purtova, 2017). Further, given that effects of aggregated data often become visible after the data has been used, a broader definition of a community may be more responsive to its members’ interests. This places emphasis on exploring, as Hick’s has argued, people’s communicative practice, and the social practices that they ascribe to. This is to give a better understanding to their choices, and the ways in which they make meaning through their actions and processes, rather than looking for solutions only through legal or ethical means (Hicks, 2023).

Thirdly, the definition of community under the NPD Report lends itself to some criticism based on how it assumes communities are formed. The definition of community presumes that communities have social or economic interests whereupon they come together (Nagaraj et al., 2021). Often these groups come together based on inherent aspects of identity such as religion or race or gender, where characteristics are ascribed to members with little or no input from them (Bhardwaj & de Souza, 2023; “Our response to the report by the committee of experts on non-personal data governance framework,” 2020). There are also groups that are based on ad-hoc relations which emerge and then dissolve for a limited period of time, for instance groups based on consumption of a particular good or service like patrons of a restaurant for a particular month (Mittelstadt, 2017). The definition provided in the NPD Report does not sufficiently engage with the fluidity around the emergence and dissolutions of communities both in terms of the rationale for their existence as well as the period for which they operate.

Fourthly, the underlying rationale of vesting a community with rights only over data which it has helped produce indicates a thinner conception of a community, and what data it might exercise rights over. A community may have an interest in data despite producing only a part of it, or despite not contributing to its collection. For example, this may include publicly or privately held climate or weather data. In these instances, the impacts of such data would accrue to a much larger segment of people than those who have immediately generated such data—a community of farmers may have an interest in such data to better anticipate and mitigate the impact of climate change over agricultural produce.

Fifthly, while the Report expressly acknowledges the need for a community to exercise rights over data to minimise harms to itself, it does not provide any insight into the nature of these harms, and how a community might be able to eliminate or minimise them. Instead, the Report’s predominant focus appears to be on securing economic rights over data, which is further evident from the Committee’s repeated articulation of data as a “natural resource”, which must be exploited for people’s material well-being (NPD Report, p. 58).

By contrast, algorithmic decision making may result in harms that are not easily identifiable or are too remote (Citron & Solove, 2021). These may be harms resulting in economic loss, discrimination, or creating a chilling effect on members of a community. Sometimes, data may be required to establish the very fact of harm. These considerations may need to be factored in when considering a framework for community data rights. For example, anonymised location information from smartphones may be used to limit timings for a public transportation system based on how people have historically used the facilities. However, different groups may be impacted differently by such a change. For instance, women may be deprived of means to travel safely from the workplace to their residence late at night, or university students may be prevented from accessing affordable transportation services early in the morning.

Sixthly, the framework for community data under the NPD Report does not provide any clarity on the relationship between the individual and the community, and how and when an individual comes to become a member of the community (Sinha & Basu, 2021). This is essential to ensure that representation of, and rights exercised by the community do not come at the cost of individual rights. For instance, platform companies organise workers by creating location hotspots (Rochmyaningsih, 2021). These are typically areas where demand for services such as ride hailing, or food delivery are very high. Based on these hotspots, companies provide workers with jobs, incentives, information about other workers, and therefore create a relationality where the aggregated data affects not just the individual driver, but also how this worker interacts with others (Chan, 2019; Kim, 2022).

While the NPD Report suggests a data trustee model to operationalise the exercise of rights by the community, the Report fails to provide any mechanism for deliberative decision making between the members of the community (Bhardwaj & de Souza, 2023), especially if the members disagree on the use or application of a particular High Value Dataset.

This section has demonstrated that regulating rights and data for a community requires one to examine how these groups are formed, who the powerful actors are within the group, how these actors make decisions within and outside the community, and what are the ways to ensure that community interests are plural. For this to occur it is important to highlight the structural inequalities that exist within a data economy (Dencik et al., 2019), and to ensure that people can actively take part and act in their own interest (Taylor, 2017). This approach places an emphasis on how communities engage with technology and if they have the autonomy to use technology in a manner that is beneficial, or opt out when it is harmful (Peña Gangadharan & Niklas, 2019; Taylor, 2017).

As we have seen within communities, there are different interests and perspectives at play. To account for these different interests necessitates asking and understanding who controls the narrative around data, what are the purposes for which data is collected, who decides such purposes, what are the ways in which it can be governed, and who are the beneficiaries (Dencik & Sanchez-Monedero, 2022). These questions contribute to studying the material effects of data as it mediates, influences, and controls behaviours in a society (Jasanoff, 2017).

These questions raise attention to challenges of justice and injustice and enables us to examine who does data leave out, what existing structures does it reinforce, and who benefits from these structures (Viljoen, 2020). Through deliberation it is possible to not just examine the implications for datafication but also how datafication creates forms and ways for economic and social organisation (Dencik et al., 2019).

It helps make explicit concerns about social justice by acknowledging challenges of inequality, or domination of some interests over others. For instance, most big tech firms are in the Global North, and therefore the priorities of these companies typically revolve around the agendas set in the locations in which they are registered or headquartered. This was typified by a recent case where Meta took a differentiated approach to linking WhatsApp and Facebook in the US, Europe and in India, and therefore offered more protections to users in the Global North (IANS, 2021). Many Indians in urban settings made a switch to Signal to protest this move, but many were compelled to stay on the platform. The change highlighted users’ differential capacity to challenge the company’s decision. The presence of robust and accessible regulatory mechanisms in the EU ensured a stronger pushback against the policy, as compared to India where in the absence of a regulatory forum, citizens’ only choice was to leave or continue using the platform.

Thinking about community data therefore also requires noting that corporations will draw on their computational powers to dominate the discourse on policy globally, using countries and communities in the South as experimenting ground (Mejias & Couldry, 2019).

These differentiated impacts and consequences of data require an approach that considers the ways in which people are made visible/invisible by data and that considers their agency to determine what degree of visibility works for them (Taylor, 2017). This would include acknowledging that data creates exclusions and inclusions in terms of how people are able to participate and make decisions for themselves about themselves (Heeks & Renken, 2018).

## How Communities See the Law and How Law Sees Communities

As we have discussed, with the emergence of proposals for community data in India, the following criticisms have emerged. The lack of a precise definition that accounts for the fluidity of communities; limitations from looking at communities primarily as entities that seek to further an economic interest and not those that are identity based; and the lack of acknowledgment of algorithmically determined communities and the challenges therefrom. In this section we explore how communities have been categorised and governed under Indian law over time and seek to identify key aspects that one can take away to govern communities prior to attempts in the data economy.

This includes various ways in which the law has recognised and tried to operationalise community rights, and the nature and extent of such rights through creating and acknowledging group interests. In addition to this, we also examine how communities have organised themselves to use existing legal tools to their advantage, even in the absence of formal rights or recognition. An analysis of these formal and informal structures highlights not just how the regulatory landscape has accommodated community interests, but also several limitations in how communities are able to access justice. This thereby offers useful lessons for building a robust regime for community rights over data.

This section looks at not just enforcement of substantive rights (minority rights), but also considers procedural fairness, which has resulted in creating mechanisms for the redressal of collective interest(s) (the case of public interest litigation). In doing so, we try to see if and how such existing and innovative frameworks for community rights can be adopted for communities to be able to exercise rights over non-personal data.

One of the most significant illustrations of the recognition of group or community rights can be found under the Indian Constitution itself. Article 25 of the Constitution confers individuals with the right to freely profess, practice and propagate their religion subject to restrictions such as public order, morality, and health. In contrast, Article 26 of confers a religious denomination, or any section thereof, with the fundamental right to establish and maintain institutions for religious and charitable purposes, to manage its own affairs in matters of religion, to own and acquire movable and immovable property, and to administer such property in accordance with law. The rights under Article 26 are also subject to the same considerations of public order, morality, and health. Therefore, while Article 25 confers individuals with the right to religious freedom, Article 26 directly confers rights on the group itself.

The right enshrined under Article 25 is subject to the state’s right to regulate or restrict any economic, financial, political, or other secular activity which may be associated with religious practice, and to bring in legislation for social welfare and reform. Similarly, the rights under Article 26 can be exercised ‘in accordance with law’.

These restrictions under Article 25, and Article 26 have led to fierce contestations to decide the nature and scope of these rights as between the community and its constituents, and vis-à-vis the state. In the context of how groups or communities identify themselves, the Indian Supreme Court has, on occasion, rejected communities’ Article 25 claims that they are of a distinct or separate religion, and therefore their practices (including their desire to exclude non-members from accessing places of worship), was constitutionally protected (Sastri Yagnapurushadji v Muldas Brudardas Vaishya). To decide such disputes, the Indian Supreme Court has adopted the ‘essential practice test’, by which the Court itself becomes the arbiter to determine whether a practice is essentially religious, or secular in nature but culturally or otherwise associated with religious practice (Bhatia, 2019). The latter includes practices relating to tenancy or succession. While a critique of this test is beyond the scope of this paper, the Court’s use of this test highlights that religious communities in India do not have a complete right to self-determination and often, their very recognition is contingent on the courts’ acceptance.

Similarly, the Supreme Court has also applied the essential practice test to determine whether a religious group has the power to excommunicate its members, that is, whether excommunication is an ‘essential’ practice of a particular religious community (Sardar Syedna Saifuddin v. State of Bombay). While the practice was initially upheld (Sardar Syedna Saifuddin v. State of Bombay), given that excommunication sometimes has consequences akin to social boycott, the Court has since expressed some doubts about the correctness of this position. The issue has recently been referred to a larger bench of the Supreme Court (Central Board of Dawoodi Bohra Community & Anr. versus The State of Maharashtra & Anr).

Therefore, in the context of religious freedoms, Indian constitutional courts have assumed the critical role in determining aspects of identity or practice of religious groups, often at the cost of how the community views itself. In the existing proposal under the NPD Report, which proposes a ‘data trustee’, a similar issue is likely to emerge, in that a supposedly neutral arbiter may ultimately decide the constituents of a community, rather than the members of the community itself. Therefore, any framework for community rights in a digitally mediated world must necessarily account for the process by which a community will come to be recognised. Further, it must also account for the remedies which can be availed of by its constituents in the event their interests or rights are adversely impacted by the actions of the community.

Another significant intervention recognising collective interest is the concept of Public Interest Litigation (PIL). The PIL is a judicial innovation by the Indian Supreme Court which arose out of a realisation that certain basic collective rights were essential for securing personal liberty and autonomy (Craig & Deshpande, 1989). It provided an opportunity for individuals to sue in representative capacity on behalf of communities or groups which ostensibly did not have the power or the means to access the justice delivery system, in furtherance of the latter’s constitutional and fundamental rights (Divan, 2016).

To allow individuals or organisations to approach constitutional courts on behalf of the affected group, the Supreme Court relaxed the procedural rules requiring a litigant to be personally affected by the act or omission in question (Mumbai Kamgar Sabha v. Abdulbhai Faizullabhai, 1976). In one of the earliest landmark decisions arising out of a PIL, the Supreme Court allowed a lawyer (suing in personal capacity) to successfully challenge incarceration of undertrial prisoners over excessively long periods of time (Hussainara Khatoon v. Home Secretary). Therefore, even though the litigant himself was not personally affected, the Court took up the cause and laid down guidelines to redress the issue. Over the years, the PIL has been used to advance fundamental rights of construction workers employed by the government, the LGBTQ community and also secure liberty of bonded labourers (PUDR v. Union of India). The PIL serves as a useful illustration to highlight how groups or communities (which are not otherwise formally recognised as groups) with a common interest have used the law to seek collective redressal.

Given that PIL has evolved as judge-made law on a case-by-case basis, there is scope to imagine the PIL as an effective tool for algorithmically formed communities, or communities with a temporary collective interest which come together to seek redressal. By way of an illustration, a group of citizens may approach the court seeking redressal against the police’s use of facial recognition technology and consequent harassment or targeting based on their presence at a public demonstration against state excesses. Here, citizens attending the demonstration would constitute a community solely because of their presence at the same place and the same time and despite the absence of any immutable common characteristics.

However, recent PIL jurisprudence also offers a cautionary tale for ensuring that interests of the community are not captured by vested interests. Bhuwania has argued how allowing ‘concerned citizens’ to sue on issues affecting several groups or communities has often resulted in the Court passing directions without giving notice to, or hearing the affected communities directly (Bhuwania, 2016). Bhuwania’s research further demonstrates how the interests of a community can sometimes become subverted even by exercises that are purportedly in public interest. For instance, he points out how in a petition lodged by a lawyer (suing in his personal capacity) assailing the government’s failure to address rising pollution in the city of Delhi, the Court issued binding directions for conversion of all auto rickshaws from petrol to Compressed Natural Gas (CNG), without considering any representation from drivers or owners of such auto rickshaws. He demonstrates how this adversely impacted auto rickshaw drivers since they were unable to afford the investment required for such conversion. Even those who could afford the conversion suffered serious economic losses due to lack of adequate infrastructure to make CNG available to such a large number of auto rickshaws.

The evolution of PIL offers two distinct lessons for any potential community rights regime in the context of data governance. First, legal and regulatory mechanisms can exist to accommodate even more transient communities whose collective interest may be temporary or limited to a particular issue or a particular use of certain data. Second, in marshalling data for public interest purposes, regulatory mechanisms must allow space for communities to be represented and raise their concerns if such public interest threatens community interests.

In conclusion, why are these examples of the ways in which law recognises and structures communities and how communities use the law important? In our view, it is important for a policy on community data to be cognizant of the ways in which the operationalising of rights within a community happen in practice. In the examples we have discussed, we have demonstrated how courts sometimes intervene, taking on a paternalistic role of determining what is good for a community. In other instances, policies do not consider the context necessary for communities to be able to participate fully even in a community centred process.

In the next section, we will reflect on ways to overcome some of these challenges by focussing on what we argue are access to justice barriers that emerge in ensuring the enforcement of rights for communities (Mittal, 2020).

## Access to (Community Data) Justice

In this paper, we have so far discussed some of the challenges that have emerged in the ways in which community data is framed in the Indian context. Our criticisms extend not just to definitional challenges of a community, but also in terms of how the NPD Report describes how a community fundamentally functions. After presenting a critique of the model of community data proposed, we argue that there is value in examining how the law sees communities, and how communities see law, and to do this, we have drawn from provisions in the Indian Constitution on minority rights, as well as from jurisprudence from the Indian Supreme Court.

In this discussion, it is apparent that with these innovations, there are plenty of challenges that emerge in operationalising community rights including aspects such as identification of belonging within a community, opportunities to participate within a community, and avenues to present grievances in situations where interests are not recognised.

Consequently, we argue that a fundamental aspect to ensuring the operationalization of community data in a meaningful way is to ensure that there is emphasis placed on ensuring that these barriers are addressed.

Over the past years, there have been several modular approaches to data governance which have sought to operationalize data from a common’s perspective (Lopez Solano et al., 2022). For instance, data trusts are frameworks that are designed to use the legal structure of a trust to manage data. In these models, parties pool together their rights over data and authorise another entity to make decisions on their behalf and for the wider public. These models would typically ensure that the trustee takes on a fiduciary responsibility to ensure that the interests of the trust are preserved (Delacroix & Lawrence, 2019). Data cooperatives on the other hand are models where individuals come together to voluntarily pool together their data so that it may benefit the group. Typically, in these models, groups come together to be able to address the challenges that emerge when because of the fragmentation of data, it becomes difficult to understand the insights about data that affect health, social and economic functions (Pentland & Hardjono, 2020). Cooperatives are arguably seen as formations that are more democratic in nature and can both represent the autonomy of the members, but also foster participation because of their voluntary and open membership (Tait, 2021). Models for indigenous data sovereignty have placed an emphasis on the collective aspects of rights. In doing so, as the CARE principles of data governance articulate, there is an emphasis on developing ways to strengthen collective benefit- through improved governance and participatory engagement of people(Carroll et al., 2020). An importance is placed on devolving authority through recognizing rights and interests of groups. In addition to this, there is an emphasis on responsibility to account for different worldviews and cultures, and commitments to ethics which are designed to account for present and future use based on the values in the community (Carroll et al., 2020).

These modular approaches are relevant to our discussion on operationalizing community data because they are offer alternative ways to organise people around data and raise novel ways of thinking in terms of membership, control, autonomy, economic benefit, and ownership (Micheli et al., 2020). They provide a basis for how community data can work in action and offer methods for how to operationalize it.

In our discussions in Sect. 3 and 4 of this paper, an important consideration was how communities are fluid entities. The manner in which they emerge and then dissolve is subject to several factors including around identity as well as interest. These in turn determine how the membership of the community emerges.

The different governance models demonstrate how data communities come together and provide pathways for how they are constructed, run, and subsequently sustained. This suggests that a key consideration is to think about the membership lifecycle a data community (Kennedy, 2018). This entails that members of a community will have different encounters within the community such as when a person becomes part of a data community; when they participate and interact as a member of the data community, and finally in terms of making decisions about their future within the data community and the future of the community itself. Taking these differences into account in the construction of a data community is significant because such communities as we have discussed earlier in the paper are not always intentional (based on identity, interest, as well as algorithmically created), and its purposes can change over time as members change.

In each of the departures identified as part of a life cycle of a member within a community, we would like to ascertain elements of access required to ensure enforcement of rights and entitlements (Cappelletti & Garth, 1981). This will allow us to trace a path to benefiting from community data for all community members. We draw from the concept of a ‘path to justice’ which can be described as the legal needs that a person requires to fulfil their requirements for a just outcome (Barendrecht et al., 2008; Genn, 1999). Drawing from this, we develop the notion of access to justice, which is the ability that people have to be able to make choices that enhance their capacity to reach outcomes which are in their interest and are fair and just (de Souza, 2022a). We view ‘access’ as a multifaceted concept from which includes aspects of ability to make one’s concerns known, opportunity to intervene in decision making that concerns one’s interest, agency, and networks to be able to participate and seek justice within a data economy (Albiston & Sandefur, 2013; Brinks, 2019).

In our view, given that communities are dynamic entities that contain an element of fluidity to their membership, we look at the question of access to justice from the perspective of the claims that are made by people individually and collectively (Ghai & Cottrell, 2009). Therefore, for the enforcement of their community data rights, we argue that it is not sufficient to just create community data institutions like data trusts, or data cooperatives, because only focusing on institutions, will not be sufficient to consider the variations of the demands made by different groups of people. We therefore advocate for thinking about access considerations at different points of time during the membership lifecycle of a person.

In the first stage at the time of joining a community, a member should have the capacity to be able to recognise that they are a part of the community. This entails that there should be awareness among members that such a group exists, that it has certain purposes for which it has been set up and that there are procedures in place through which governance takes place within the group. Such a recognition involves access to information and knowledge wherein the member can interrogate the antecedents of the community and whether it exists for the collective benefit of all in the group. As we have discussed, communities are both intentionally as well as unintentionally created, and therefore if we wish to ensure that there is access to justice, a core component is to ensure that members are able to have avenues to represent their views and take actions in their benefit.

In the second stage when members are part of community, it becomes imperative that they are able to examine the internal hierarchies in the community and have the capacity to be able to contest decisions through open and deliberative channels (Kukutai & Taylor, 2016). This ensures that being a part of a community also involves being able to challenge interests of the group, as well as contribute to its development (Walter et al., 2020). Community data reflects structural challenges that already exist in a society. As a result, certain worldviews are privileged over others, making it imperative for members to also ensure that their points of views are not subsumed in the epistemic choices being made by others in the group.

To ensure participation within a community, a cornerstone of access to justice, it is imperative to account for the structural ways in which community relations arise as discussed in Sect. 3, decisions that emerge as a consequence of internal community dynamics, and finally the procedural aspects that determine how communities can be governed as demonstrated in Sect. 4. As examples from how minority rights are defined and how public interest litigation is filed shows, community interest needs to be continuously remembered, renegotiated, and reconstituted, as they change dynamically and fluidly (Costanza-Chock, 2020).

Therefore, participation within a community is a regular process of continual reflection and renewal of interest. In this regard, ensuring that members can voice and make visible their concerns, and have agency to drive the purpose of the community is a key component of participating within a community (Gurumurthy & Chami, 2022).

In the third stage, if communities do not work for its members, it is imperative that there is an exit option available to members who no longer wish to be part of a community. In the examples of public interest litigation discussed in the previous section, where for instance the voice of community becomes secondary to the life of the case itself or in cases of religious communities where individuals can be excommunicated, avenues for redressal of grievance are necessary.

In this instance, members should be able to determine the future of the community and ensure that it continues to work in their interest. In cases where there is conflict, there should be mechanisms to account for differences, but also opportunities to opt out of such arrangements when the collective interest or benefit is to the detriment of individual interests. (Manohar et al., 2020). We think that these factors are important particularly to ensure that access to (community data) justice is meaningful and able to respond to the complexity of communities.

This section has proposed developing an approach that places an emphasis on the access challenges that can emerge for members within a data community. It offers reflections on what kinds of considerations should be kept in mind to ensure that members are able to participate effectively, and that their interests are met. In doing so, we offer pathways towards how to ensure that community data is reflective as well as representative of the members in the community, and that their diverse interests and identities are understood and acknowledged.

## Conclusion

In this paper, we have sought to provide insights from discussions on community data in the Indian context. It has aimed to map key debates which describe how the concept of community data has emerged and has subsequently provided an overview of some of the critiques that would be important to keep in mind to ensure that community data can be operationalized in a meaningful way.

The paper argues that the notion of communities in law is not something that is new but that there is value in drawing from other literature and in this case, we draw particularly from rights of minorities as well as public interest litigation to demonstrate the ways in which communities see law and law sees communities. The purpose of doing so is to demonstrate that in the entanglements between law and communities there are several dynamic frictions that take place. This includes the challenge of ensuring that the interests of communities continue to be represented, that there remains a sense of belonging for communities and that they can participate and act in their own interests both individually and collectively.

The paper thereafter addresses some of these challenges that emerge in the everyday interactions between law and communities and proposes that it is important to not just focus on institutions that can enable the securing of rights around community data but rather to focus on the ways in which individuals make claims to benefits that accrue to them. This is why, in the final section of this paper we propose the idea of what access to (community data) justice could look like. In this approach we argue that it is important to explore the membership within a community and thereby examine what are the ways in which a community functions. To do this we have proposed to look at challenges at the time at which a community is formed, questions of participation within the community, as well as when members leave communities. In doing so, we hope that this contribution provides not just an understanding of the rich and nuanced challenges that emerge through the novel framework to frame community data in the Indian context but also offers strategic and practical pathways to ensure that community data can be operationalized.

## Data Availability

Not applicable.
